# Regulation of TXB_2_ and PGE_2_ production by TGF-β_1_ in *in vitro* silica dust-exposed rat alveolar macrophage

**DOI:** 10.1155/S0962935195000664

**Published:** 1995-11

**Authors:** Urszula Orlinska, Douglas C. Kuhn

**Affiliations:** 1 Department of Pathology Hershey Medical Center The Pennsylvania State University P.O. Box 850 Hershey PA 17033 USA; 2 Paradigm Biosciences, Inc. 2401 Foothill Dr. Salt Lake City UT 84109 USA

## Abstract

We investigated the effect of transforming factor factor-β_1_ (TGF-β_1_) on thromboxane B_2_ (TXB_2_) and prostaglandin E_2_ (PGE_2_) production in *in vitro* silica dust-exposed rat alveolar macrophages (AM). In the presence of 5 μg of anti-TGF-β_1_ antibodies, TXB_2_ production decreased, but PGE_2_ production increased. Addition of 2 ng of TGF-β_1_ to the culture medium potentiated TXB_2_ production, but PGE_2_ production apparently did not change. At 50 ng of TGF-β_1_, TXB_2_ production decreased, and PGE_2_ production varied. Our data suggest that in rat AM: (1) both endogenous and exogenous TGF-β_1_ regulate TXB_2_ production; and (2) in the absence of endogenous TGF-β_1_ the liberation of PGE_2_ increases; however, exogenous TGF-β_1_ does not have a regulatory effect on PGE_2_.


Mediators of Inflammation 4, 413–416 (1995)

### Regulation of $TXB_2$ and $PGE_2$ production by TGF-$\beta_1$ in *in vitro* silica dust-exposed rat alveolar macrophage

Urszula Orlinska[CA,*] and Douglas C. Kuhn

Department of Pathology, Hershey Medical Center, The Pennsylvania State University, P.O. Box 850, Hershey, PA 17033, USA
*Present address: Paradigm Biosciences, Inc., 2401 Foothill Dr., Salt Lake City, UT 84109, USA

[CA]Corresponding Author

**We investigated the effect of transforming factor factor-$\beta_1$ (TGF-$\beta_1$) on thromboxane $B_2$ ($TXB_2$) and prostaglandin $E_2$ ($PGE_2$) production in *in vitro* silica dust-exposed rat alveolar macrophages (AM). In the presence of 5 µg of anti-TGF-$\beta_1$ antibodies, $TXB_2$ production decreased, but $PGE_2$ production increased. Addition of 2 ng of TGF-$\beta_1$ to the culture medium potentiated $TXB_2$ production, but $PGE_2$ production apparently did not change. At 50 ng of TGF-$\beta_1$, $TXB_2$ production decreased, and $PGE_2$ production varied. Our data suggest that in rat AM: (1) both endogenous and exogenous TGF-$\beta_1$ regulate $TXB_2$ production; and (2) in the absence of endogenous TGF-$\beta_1$ the liberation of $PGE_2$ increases; however, exogenous TGF-$\beta_1$ does not have a regulatory effect on $PGE_2$.**

**Key words:** Rat alveolar macrophages, $PGE_2$, TGF-$\beta_1$, $TXB_2$

### Introduction

Chronic airway inflammation and lung fibrosis are common features of prolonged exposure to mineral particles of silica in humans and in rodents.[1] Alveolar macrophages (AM) have an essential role in lung clearance and defence mechanisms against inhaled particles.[2] They fulfil these functions via inflammatory mediators produced upon macrophage activation. Among the many mediators are arachidonic acid metabolites such as thromboxane $A_2$ ($TXA_2$) and prostaglandin $E_2$ ($PGE_2$). Thromboxane $A_2$ (of which $TXB_2$ is the stable metabolite) is a very potent platelet aggregating factor and pulmonary vasoconstrictor. A recent study of the regulatory effect of $TXB_2$ on the proliferation of vascular smooth muscle cells from rats demonstrated a rapid build-up of cytoskeletal protein in these cells in hypertension, suggesting that $TXB_2$ may play a role in the remodelling of the vascular wall in hypertension.[3,4]

Prostaglandin $E_2$ is one of the key inflammatory mediators with multiple functions. In the lung, it induces bronchodilation and causes an increase in epithelial cell chloride secretion.[5] $PGE_2$ induces vasodilatation in unventilated foetal lung.[6]

Another mediator that is produced by alveolar macrophages is transforming growth factor-$\beta_1$ (TGF-$\beta_1$). TGF-$\beta_1$ is a multifunctional peptide. TGF-$\beta_1$ can influence cell proliferation or differentiation and other functions in various types of cells, some of which are related to inflammation and tissue fibrosis. For example, TGF-$\beta_1$ increases the rate of fibronectin gene transcription,[7] can down-regulate interleukin-1β receptors,[8] and inhibits interleukin-6 release from fibroblasts.[9] The most profound effect of TGF-$\beta_1$ is its ability to enhance the formation of extracellular matrix. TGF-$\beta_1$ promotes the formation of collagen and fibronectin in fibroblasts of human, rat and mouse origin. From *in vivo* studies, it has been shown that TGF-$\beta_1$ can stimulate the formation of the typical granulation tissue found in tissue repair.

Although pulmonary macrophage-derived mediators have a pivotal role in lung inflammation and fibrosis, the interrelationship between these mediators is just beginning to be understood. We have shown the very early release (15 min) of TGF-$\beta_1$ from silica dust-exposed rat alveolar macrophages.[10] The amount of TGF-$\beta_1$ released at this time was the greatest over 24 h kinetic studies, and preceded the production of $TXB_2$ and $PGE_2$. To further unravel the interrelationship between eicosanoids and TGF-$\beta_1$ produced by alveolar macrophage, the modification of $TXB_2$ and $PGE_2$ production by TGF-$\beta_1$ in *in vitro* silica-exposed rat alveolar macrophages was investigated. We observed decreased production of $TXB_2$ in the presence of anti-TGF-$\beta_1$ antibodies, or high concentration (50 ng) of TGF-$\beta_1$. $TXB_2$ production increased in the presence of a low concentration of TGF-$\beta_1$ (2 ng) in the culture medium. $PGE_2$ production increased in

© 1995 Rapid Science Publishers the presence of anti-TGF-$\beta_1$ antibodies. Exogenous TGF-$\beta_1$ at low or high concentration did not have any effect on $PGE_2$ production in rat alveolar macrophages.

### Materials and Methods

*Materials:* Hanks' solution and Dulbecco's phosphate buffered saline (DPBS), and HEPES (4-(2-hydroxyethyl)-1-piperazine ethanesulfonic acid) buffer were prepared in our laboratory. Six-well tissue culture plates were obtained from Falcon Laboratory Products (Beckton and Dickinson Labware, Lincoln Park, NJ). DMSO (dimethylsulfoxide) was obtained from Calbiochem (San Diego, CA). Allopurinol, oxypurinol, allopurinol riboside, Penn/Strep, M199 medium (without phenol red), and human serum albumin were all obtained from Sigma (St. Louis, MO). Human recombinant and polyclonal anti-TGF-$\beta_1$ antibody were obtained from R&D Systems (Minneapolis, MN). RIA reagents for $TXB_2$ and $PGE_2$ assay were purchased from Advanced Magnetics, Inc. (Boston, MA).

*Isolation and culture of rat alveolar macrophages:* Sprague–Dawley rats weighing approximately 225 g were lavaged *ex vivo*. Rats were given an intramuscular injection of sodium pentabarbital (10 mg) and the rib cage was opened to expose the lungs. An incision halfway through the trachea was made and the lavage tube of the trachea cannula was inserted into the incision. The lungs were washed five times each with 10 ml of lavage fluid (phosphate-buffered saline containing 0.1% EDTA, at 37°C). The collected lavage fluid was filtered through sterile Nitex gauze and centrifuged at 250 × **g** for 10 min. The supernatant was discarded, and the cells resuspended in M199, 10 mM HEPES 0.3% human serum albumin, 100 U/ml penicillin and 100 μg/ml streptomycin (culture medium). Cells from all rats were pooled and centrifuged again at 250 × **g** for 10 min. Cells were enumerated in a haemocytometer. Viability was determined by the exclusion of trypan blue and cells were plated at $1 \times 10^6$/ml of culture medium in 6-well culture plates. The cells were preincubated for 1 h at 37°C in a humidified atmosphere at 5% $CO_2$ in air. The nonadherent cells were removed by aspiration. Fresh culture medium (37°C) was placed in the wells and unlabelled arachidonic acid was added at a final concentration of 3 μM.[10] This incubation with arachidonic acid was conducted in order to replenish endogenous arachidonic acid utilized during the exaggerated eicosanoid production which occurs during cell isolation and attachment.[11] The cells were incubated at 37°C for 24 h. After incubation the medium containing arachidonic acid was removed and fresh culture medium was placed in the wells. Macrophages were activated with a microcrystalline form of silica dust (Min-U Sil, 2–7 μm diameter, provided by Dr Peter Bolsitis, MIT) at a final concentration of 200 μg/ml, at 37°C for 24 h, in the absence or presence of anti-TGF-$\beta_1$ antibodies, or TGF-$\beta_1$. Nonspecific antibody as an isotype-matched control for anti-TGF-$\beta_1$ antibody was tested. Media were collected into silianized tubes for the determination of eicosanoids.

*Radioimmunoassay for $TXB_2$ and $PGE_2$:* Following incubation, the culture medium was removed and acidified to pH 3.0 with 1 N HCl. Eicosanoids were extracted twice with 2 ml ethyl acetate/cyclohexane (1:1). The extracts were stored at −20°C until analysis of eicosanoids by specific radioimmunoassay, as described previously.[12] The antibodies utilized in the assay of $TXB_2$ or $PGE_2$ showed less than 1% cross-reactivity with other eicosanoids.

*Cell viability test:* The lactate dehydrogenase (LDH) activity in rat culture conditions was measured using a Sigma kit for LDH. There was no detectable LDH enzyme activity in the culture medium from rat alveolar macrophages.

*Data analysis:* Results are presented as means ± S.E. of a number of independent experiments.

### Results

*The effect of TGF-$\beta_1$ on $TXB_2$ production in rat alveolar macrophages:* Table 1 shows the effect of anti-TGF-$\beta_1$ antibodies, or low or high concentrations of TGF-$\beta_1$, on $TXB_2$ production in silica-exposed rat alveolar macrophages. In the presence of 5 μg of anti-TGF-$\beta_1$ antibodies in the culture medium, during the process of macrophage activation with silica, the production of $TXB_2$ was inhibited. The TGF-$\beta_1$, at 2 ng concentration in the culture medium, potentiated $TXB_2$ production. At 50 ng of TGF-$\beta_1$ in the culture medium, the production of $TXB_2$ was inhibited.

*The effect of TGF-$\beta_1$ on $PGE_2$ production in rat alveolar macrophages:* Table 2 shows the effect of anti-TGF-$\beta_1$ antibodies, or low or high concentrations of TGF-$\beta_1$, on $PGE_2$ production in silica-exposed rat alveolar macrophage. In the presence of 5 μg of anti-TGF-$\beta_1$ antibodies in the culture medium, the production of $PGE_2$ was

**Table 1.** The effect of TGF-$\beta_1$ on $TXB_2$ production by rat alveolar macrophages exposed to silica dust

| Treatment | Experiment 1 | | Experiment 2 | | Experiment 3 | |
|---|---|---|---|---|---|---|
| | $TXB_2$ (pg/ml) | % Control | $TXB_2$ (pg/ml) | % Control | $TXB_2$ (pg/ml) | % Control |
| Control | $62 \pm 2$ | | $179 \pm 5$ | | $79 \pm 3$ | |
| Silica | $132 \pm 2$ | 213 | $369 \pm 2$ | 206 | $172 \pm 9$ | 218 |
| Silica + 5 µg anti-TGF-$\beta_1$ | $60 \pm 5$ | 100 | $313 \pm 2$ | 175 | $103 \pm 7$ | 130 |
| Silica + 2 ng TGF-$\beta_1$ | ND | ND | $491 \pm 3$ | 274 | $219 \pm 2$ | 277 |
| Silica + 50 ng TGF-$\beta_1$ | $47 \pm 5$ | 76 | $69 \pm 0$ | 39 | $158 \pm 3$ | 200 |

Alveolar macrophages at $1 \times 10^6$ cells were activated with 200 µg of silica dust in the absence or presence of anti-TGF-$\beta_1$ antibodies, or TGF-$\beta_1$ added at the time of cell activation, and incubated at 37°C for 24 h. $TXB_2$ was assayed by the RIA as described in Methods. $n = 2$ in each experimental group. ND = not done.

**Table 2.** The effect of TGF-$\beta_1$ on $PGE_2$ production by rat alveolar macrophages exposed to silica dust

| Treatment | Experiment 1 | | Experiment 2 | | Experiment 3 | |
|---|---|---|---|---|---|---|
| | $PGE_2$ (pg/ml) | % Control | $PGE_2$ (pg/ml) | % Control | $PGE_2$ (pg/ml) | % Control |
| Control | $445 \pm 6$ | | $207 \pm 9$ | | $411 \pm 10$ | |
| Silica | $939 \pm 3$ | 211 | $241 \pm 10$ | 116 | $438 \pm 10$ | 107 |
| Silica + 5 µg anti-TGF-$\beta_1$ | $1137 \pm 75$ | 256 | $330 \pm 9$ | 159 | $783 \pm 20$ | 191 |
| Silica + 2 ng TGF-$\beta_1$ | ND | ND | $253 \pm 3$ | 122 | $457 \pm 2$ | 111 |
| Silica + 50 ng TGF-$\beta_1$ | $840 \pm 91$ | 189 | $227 \pm 7$ | 110 | $504 \pm 20$ | 123 |

Alveolar macrophages at $1 \times 10^6$ cells were activated with 200 µg of silica dust in the absence or presence of anti-TGF-$\beta_1$ antibodies, or TGF-$\beta_1$ added at the time of cell activation, and incubated at 37°C for 24 h $PGE_2$ was assayed by the RIA as described in Methods. $n = 2$ in each experimental group. ND = not done.

potentiated. The addition of 2 ng of TGF-$\beta_1$ to the culture medium had no apparent effect on $PGE_2$ production in either of two experiments performed. At 50 ng of TGF-$\beta_1$, the production of $PGE_2$ varied.

### Discussion

This study was designed to evaluate the regulation of $TXB_2$ and $PGE_2$ production by TGF-$\beta_1$ in alveolar macrophages. The reason for these studies was to supplement the limited information in the literature on the interrelationship between the inflammatory mediators released by a specific inflammatory cell. $TXB_2$, $PGE_2$ and TGF-$\beta_1$ are the principal inflammatory mediators released by alveolar macrophages during the lung's defence process against pathogens or foreign matter. We have demonstrated in earlier studies[10] that these three mediators are released from rat alveolar macrophages upon exposure to silica dust. In those studies we observed a striking difference between the kinetics of release of TGF-$\beta_1$ (maximum release at 15 min post-silica dust) and $TXB_2$ (plateau release at 6 h post-silica dust), or $PGE_2$ release (plateau release at 6 h post-silica dust). Because it is thought that TGF-$\beta_1$ has a central regulatory role in vascular physiology and pathology, we formulated a hypothesis that perhaps TGF-$\beta_1$ has an impact on the production of either $TXB_2$ or $PGE_2$, or both, by alveolar macrophage upon silica dust-exposure.

We observed a positive effect of TGF-$\beta_1$ on $TXB_2$ production in rat alveolar macrophages which had been exposed to silica dust. In the absence of TGF-$\beta_1$ (plus anti-TGF-$\beta_1$ antibodies), the quantity of $TXB_2$ released from rat alveolar macrophages to the culture medium in response to silica exposure was significantly smaller when compared to the response with silica alone. Exogenous TGF-$\beta_1$, at low concentration, potentiated the action of silica dust, and the amount of $TXB_2$ produced by rat alveolar macrophages was significantly greater than the amount of $TXB_2$ released by alveolar macrophages activated by silica alone. High concentration of TGF-$\beta_1$, however, inhibited $TXB_2$ production in rat alveolar macrophages; in response to silica, the amount of $TXB_2$ released was smaller than the amount of $TXB_1$ produced in rat alveolar macrophages activated with silica alone, or in the absence of TGF-$\beta_1$ (in the presence of anti-TGF-$\beta_1$ antibodies). This observation suggests that upon exposure to silica dust a homeostatic autocrine loop for TGF-$\beta_1$, that regulates the production of $TXB_2$, appears to function in rat alveolar macrophages. Our data also suggest the role for concentration of TGF-$\beta_1$ in the regulation of $TXB_2$ production; depending on the concentration, this growth factor appears to be either stimulatory or inhibitory to rat alveolar macrophage, and to $TXB_2$ production.

At the same time, however, the effect of TGF-$\beta_1$ on the generation of another eicosanoid, namely $PGE_2$, in silica-exposed rat alveolar macrophages appeared to be very different to that observed for $TXB_2$. In the absence of TGF-$\beta_1$ (plus anti-TGF-$\beta_1$ antibodies), the amount of $PGE_2$ produced in silica-exposed alveolar macrophages was greater than when compared to silica alone. When anti-TGF-$\beta_1$ antibodies were present in the culture medium during the exposure of macrophages to silica, we observed that (1) these macrophages liberated greater amount of $PGE_2$ compared to silica alone; and (2) there was a stimulation of $PGE_2$ production by alveolar macrophages which did not produce $PGE_2$ after exposure to silica. Furthermore, the presence of exogenous TGF-$\beta_1$, at low or high concentrations, had either no apparent effect, or a variable effect, respectively, on $PGE_2$ production in silica-exposed rat alveolar macrophages when compared to silica alone. Our data suggest, therefore, a possible distinct biological role for endogenous, but not exogenous TGF-$\beta_1$ to down-regulate $PGE_2$ production in rat alveolar macrophages after exposure to silica.

Data presented in this manuscript provide evidence of the regulatory role of TGF-$\beta_1$ in the liberation of inflammatory eicosanoids, $TXB_2$ and $PGE_2$, in rat alveolar macrophages, when the cells are exposed to silica dust. In support of our observation are the reports of Datta *et al.*, where the authors demonstrate that TGF-$\beta_1$ inhibits lipoxygenase and epoxygenase eicosanoid production by osteosarcoma cells,[13] or eicosanoid metabolism in osteogenic osteosarcoma cells.[14] The regulation of eicosanoid liberation by TGF-$\beta_1$, or the regulation of TNF-$\alpha$ production by TGF-$\beta_1$[13] suggests that the mechanism of action of TGF-$\beta_1$ on tissues may not necessarily be direct, but rather it requires actions of other mediators. The interrelationship between TGF-$\beta_1$ itself and all the inflammatory mediators directed by TGF-$\beta_1$ in inflammation appears to be very intriguing.

In summary, our data suggest that in silica-exposed alveolar macrophages, both endogenous and exogenous TGF-$\beta_1$ regulate $TXB_2$ production. Our data suggest that endogenous, but not exogenous TGF-$\beta_1$ has a regulatory role in $PGE_2$ production. Because both $TXB_2$ and $PGE_2$ were measured in the same samples, and because there is a consistent increase in silica-stimulated $PGE_2$ synthesis in the presence of anti-TGF-$\beta_1$ antibodies in the absence of an effect of authentic TGF-$\beta_1$, it appears that in silica-exposed alveolar macrophages the regulatory effects of TGF-$\beta_1$ are different between the different components of the arachidonic acid pathway.

### References

1. Davis GS. Pathogensis of silicosis: current concepts and hypotheses. *Lung* 1986; **164:** 139–154.
2. Tarling J, Lin HS, Hsu S. Self-renewal of pulmonary alveolar macrophages: evidence from radiation chimera studies. *J Leukocyte Biol* 1987; **42:** 443–446.
3. Ishimutsu T, Uehara Y, Ishii M, Ikeda T, Matsuoka H, Sugimoto T. Thromboxane and vascular smooth muscle cell growth in genetically hypertensive rats. *Hypertension* 1988; **12:** 46–51.
4. Nagata T, Uehara Y, Numabe A, *et al.* Regulatory effect of thromboxane $A_2$ on proliferation of vascular smooth cells from rats. *Am J Physiol* 1992; **263:** H1331–H1338.8.
5. Sigal E. The molecular biology of mammalian arachidonic acid metabolism. *Am J Physiol* 1991; **260:** L13–L28.
6. Cassin S, Tod M, Philips J, Frisinger J, Jordan J, Gibbs C. Effects of prostaglandin $D_2$ on perinatal circulation. *Am J Physiol* 1981; **240:** H755–H760.
7. Dean DC, Newby RF, Burgois S. Regulation of fibronectin biosynthesis by dexamethasone, transforming growth factor β, and cAMP in human cell lines. *J Cell Biol* 1988; **106:** 2159–2170.
8. Dubois CM, Ruscetti FW, Palaszynski EW, Falk LA, Oppenheim JJ, Keller JR. Transforming growth factor β is a potent inhibitor of interleukin 1 (IL-1) receptor expression: proposed mechanism of inhibition of IL-1 action. *J Exp Med* 1990; **172:** 737–744.
9. Elias JA, Lentz V, Cummings PJ. Transforming growth factor-β regulation of IL-6 production by unstimulated and IL-1 stimulated human fibroblasts. *J Immunol* 1990; **146:** 3437–3443.
10. Orlinska U, Palmer C, Kuhn DC, Demers LM. Interrelationship between hydroxyl radical formation, and thromboxane $B_2$ and transforming growth factors $\beta_1$ production in *in vitro* silica dust-exposed rat alveolar macrophage. *Am Rev Resp Dis* (accepted).
11. Kouzan S, Nolan RD, Fournier T, Bignon J, Eling TE, Brody AR. Stimulation of arachidonic acid metabolism by adherence of alveolar macrophages to a plastic substrate. *Am Rev Resp Dis* 1988; **137:** 38–43.
12. Demers LM. Prostaglandins, thromboxane, and leukotrienes. In Race GJ, ed. *Laboratory Medicine.* Philadelphia: Harper and Row, 1983. pp. 1–21.
13. Datta H, Sullivan M, Ohri S, MacIntyre I. Transforming growth factor-$\beta_1$ inhibits lipoxygenase and epoxygenase eicosanoid production by osteosarcoma cells. *Biochem Soc Trans* 1990; **18:** 1250–1260.
14. Datta HK, Sullivan M, Rathod H, MacIntyre I. Transforming growth factor-β modulates eicosanoid metabolism in osteogenic osteosarcoma cells. *Biochem Biophys Res Comm* 1991; **178:** 940–945.

ACKNOWLEDGEMENTS. Work presented in this manuscript was supported by a grant from the US Dept. of Interior's Mineral Institute Program administered through the Generic Mineral Technology Center for Respirable Dust, grant number 1135142. We thank Stephen Bobar for his great help with lavages of rat lungs and for the excellent performance of radioimmunoassays.

**Received 4 July 1995; accepted in revised form 31 August 1995**
